# Immediate Effects of Diaphragmatic Breathing with Cervical Spine Mobilization on the Pulmonary Function and Craniovertebral Angle in Patients with Chronic Stroke

**DOI:** 10.3390/medicina57080826

**Published:** 2021-08-16

**Authors:** Ho Jung An, A Yeon Kim, Shin Jun Park

**Affiliations:** 1 Department of Physical Therapy, Dongnam Health University, 50, Cheoncheon-ro 74beon-gil, Jangan-gu, Suwon-si 16328, Gyeonggi-do, Korea; 0628jjang@hanmail.net; 2 Department of Physical Therapy, Graduate School, Yongin University, 134, Yongindaehak-ro, Cheoin-gu, Yongin-si 17092, Gyeonggi-do, Korea; wkdskfktkrhk@hanmail.net; 3 Department of Physical Therapy, Suwon Women’s University, 1098, Juseok-ro, Bongdam-eup, Hwaseong-si 18333, Gyeonggi-do, Korea

**Keywords:** diaphragmatic breathing, joint mobilization, pulmonary function, stroke, CVA

## Abstract

*Background and Objectives*: Patients with stroke have a forward neck posture due to neurological damage and often have impaired pulmonary function. This study investigated the effect of diaphragmatic breathing with cervical mobilization to improve pulmonary function cervical alignments. *Materials and Methods*: This study used a one-group pre-test–post-test design including 20 patients with stroke. Two types of cervical joint mobilization techniques, consisting of left and right lateral glide mobilization and posterior–anterior mobilization, were utilized. During joint mobilization, the patients performed diaphragmatic breathing. The measurements were performed immediately after the intervention. Pulmonary function was evaluated using a spirometer to measure the forced expiratory volume in 1 s (FEV1), forced vital capacity (FVC), and peak expiratory flow (PEF). The craniovertebral angle (CVA) was measured using lateral photographs. *Results*: After diaphragm breathing with cervical joint mobilization, subjects had significantly increased FEV1, FVC, PEF and CVA. *Conclusion*: Diaphragm breathing with cervical joint mobilization are possible interventions to increase pulmonary function and improve the craniovertebral angle in patients with stroke. However, a complete conclusion can be reached only after a follow-up study has been conducted with a comparison of more subjects and controls.

## 1. Introduction

Stroke patients have respiratory disturbances due to weakening of the diaphragm, responsible for inhalation, and the abdominal muscles, responsible for exhalation [1,2,3]. The nature of these disorders can cause abnormalities in respiratory mechanics and abnormal gas exchange, which can lead to respiratory complications [4,5]. Respiratory complications experienced by stroke patients include sleep apnea, venous thromboembolism, swallowing abnormalities, aspiration, and pneumonia [6].

In the case of patients with stroke, respiratory rehabilitation is necessary for the inhalation muscles because the more difficult it is for a patient to walk, the worse the effect on inhalation muscles is [7,8]. Threshold inspiratory muscle training was used as a method to improve inspiratory function in stroke patients [9]. This exercise is a way to improve inspiratory ability by using the diaphragm as well as accessory muscles of inspiration. Unlike threshold inspiratory muscle training, the diaphragm exercise relaxes the shoulders, head and neck, allowing only the rise of the abdomen while inhaling deeply through the nose [10].

Therefore, the importance of diaphragm exercise has been emphasized in respiratory rehabilitation for patients with stroke [11,12,13,14]. Diaphragm exercise is a method that improves the ability to inhale by strengthening the diaphragm muscles [10]. This exercise method has been proven to be capable of increasing pulmonary function and respiratory muscle strength in patients with stroke in several studies [11,12,13,14].

Diaphragm exercise is proposed to increase diaphragm contraction in patients with stroke. However, patients with stroke often have a forward neck posture due to a failure to adjust their posture [15], which can cause entrapment of the phrenic nerve [16,17,18]. Maitland joint mobilization is an manual orthopedic treatment method that uses brick wall theory as a basic concept [19]. It is a technique that applies movement to painful joints or those with limited movement [19]. This method involves passive movements applied by a therapist and is safe because the patient can stop mobilization at any time if uncomfortable when applying this approach. Maitland joint mobilization applied to the cervical vertebra reduces upper limb muscle tone and stiffness in patients with stroke [20], and when applied to the rib cage, it increases respiratory muscle tone and lung function [21,22]. In addition, manual correction applied to the cervical vertebra increases the inhalation and exhalation volumes in healthy people [23]. Although diaphragm exercise and cervical joint mobilization for diaphragm contraction in patients with stroke provide well-suited interventions for improving pulmonary function, the effects of these two methods have not yet been confirmed.

Therefore, the purpose of this study was to investigate the effects of diaphragm exercise and cervical joint mobilization in improving pulmonary function and the craniovertebral angle in stroke patients.

## 2. Methods

### 2.1. Design

This study was conducted as a one-group pre-test–post-test of quasi-experimental design. This study was conducted in accordance with the ethical principles of the Declaration of Helsinki and was approved by the institutional review board of Yong In University (No: 2-1040966-AB-N-01). Informed consent was obtained from all subjects before the pre-test. The pre-test was a pulmonary function and cervical alignments. Intervention was diaphragmatic breathing with cervical joint mobilization for stroke patients. The post-test was immediate changes in pulmonary function and cervical alignment after intervention.

No subjects had ever experienced cervical joint mobilization and were informed that this study was investigating the effects of respiratory function and cervical alignments. Subjects could not see the CVA lateral photographs, or FVC, FEV1, or PEF displays; thus, they were not provided feedback on the assessment. The evaluator, a person unrelated to this study, measured the pre-test and post-test in an independent space without knowing that this study was investigating the therapeutic effect of joint mobilization. Physical therapists who applied cervical joint mobilization were not informed that this study would confirm the effect of this procedure on respiratory function.

First, the experimental group was educated on diaphragmatic breathing for 30 min. Then, one physical therapist applied two types of cervical joint mobilization techniques (lateral sliding mobilization and anterior–posterior mobilization) for 18 min. During cervical joint mobilization, the subject was asked to perform diaphragmatic breathing. The data were collected through assessment immediately after intervention.

### 2.2. Sample Size Calculation

In this study, the sample size was calculated based on calculations through G-power 3.1.9 software (Heinrich Heine University, Dusseldorf, Germany) according to a t test of Cohen’s d [24]. Prior to calculating the total sample size, a pilot study was conducted on 6 stroke patients. According to the pilot study, the effect sizes were 1.33 (FEV1), 1.38 (FVC), 0.77 (PEF), and 0.89 (CVA). The total sample sizes was 7 (FEV1), 7 (FVC), 16 (PEF), and 12 (CVA) with each effect size; the power of the test was 0.80; and an alpha error probability of 0.05 were input parameters. The total sample size of this study was 20.

### 2.3. Participants and Recruitment

In this study, among adult patients hospitalized for stroke under the care of a rehabilitation medicine doctor at a rehabilitation hospital located in Gyeonggi-do Province, those whose diagnosis was made more than 6 months previously, those whose forced vital capacity was 10% lower than the normal predicted value [25], those with a craniovertebral angle of less than 50° [26], those who scored more than 24 points in the Korean mini-mental state examination [27], those with no given comorbidities, and those who expressed their willingness to participate in this study were selected as subjects (*n* = 20). All subjects who participated in this study were receiving a neurodevelopmental treatment (NDT) program from a physical therapist after hospitalization. The NDT programs typically included mat and gait training.

### 2.4. Intervention

#### Diaphragmatic Breathing with Joint Mobilization

**Figure 1:**
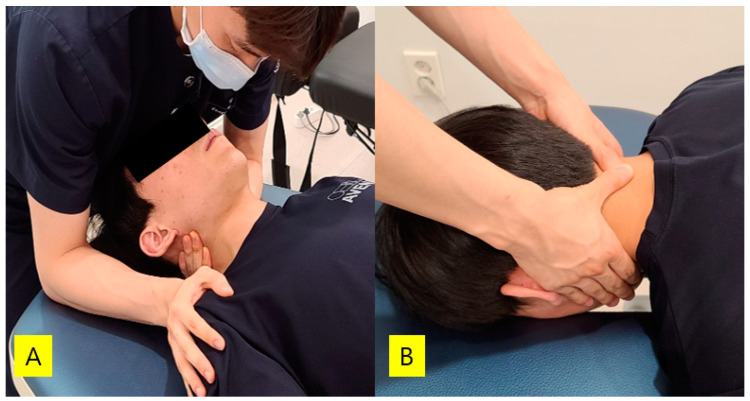
Two type of cervical joint mobilization techniques: (**A**) lateral glide mobilization; (**B**) posterior–anterior mobilization.

Diaphragmatic breathing with cervical joint mobilization was performed with the patient in a supine position. Grade Ⅲ cervical joint mobilization was applied according to the Maitland classification, and during joint mobilization, the patients performed diaphragmatic breathing with controls on breathing [10]. One of the two type of cervical joint mobilization techniques (left and right lateral glide mobilization, posterior–anterior mobilization) was performed for 1 min per set, for a total of three sets [28]. Specifically, cervical joint mobilization took a total of 9 sets of 9 min, with a rest period of 1 min between sets. For two types of cervical mobilization, left and right lateral glide mobilization and posterior–anterior mobilization were performed on the C4-5 segment (Figure 1). During left glide lateral mobilization, the physiotherapist fixed the subject’s head using the chest, and the shoulder was fixed to prevent the shoulder from rising with the opposite hand while pulling the side surrounding the cervical vertebra using the index finger of one hand [29]. The right glide lateral mobilization was applied in the same way as the left glide lateral mobilization technique. Posterior–anterior mobilization was performed by the physiotherapist standing above the head of the patients, placing the thumb of one hand on the spinous process, supporting it with the thumb of the other hand, and extending the arm in a vertical straight line [19].

### 2.5. Assessments

#### 2.5.1. Pulmonary Function

A spirometer (MicroLab spirometer ML3500 MK6, Kent, UK) was used to evaluate pulmonary function. The study subjects sat on a chair with a backrest, looked to the front, inhaled as much as possible, and then exhaled air through a spirometer for 6 s. Forced expiratory volume in 1 s, forced vital capacity, and peak expiratory flow were selected as the measurement variables. All items were measured three times and the average value was recorded. A spirometer was used to measure lung function. Pulmonary function measurements were conducted according to ATS/ERS Standardization of Spirometry guidelines for pulmonary function testing [30].

#### 2.5.2. Craniovertebral Angle

ImageJ (National Institute of Health, NIH Version, 1.32J, Bethesda, ML, USA) software for Windows was used to measure the cervical alignment in stroke patients. The study subjects sat on a chair with a backrest. Stickers were attached to the tragus of the ear and the seventh spinous process of the cervical vertebra to obtain the angle between a horizontal line and the line between these two points [31]. Lateral photographs were taken with a camera positioned vertically with the camera lens 80 cm away from the shoulder using a smartphone (iPhone, Apple Inc., Cupertino, CA, USA).

### 2.6. Statistical Analyses

The data collected in this study were statistically analyzed using the SPSS 21.0 program (SPSS Inc., Chicago, IL, USA). The Shapiro–Wilk test confirmed whether the distribution followed a normal distribution. The Cohen’s d effect size was obtained using the G * power program. A paired *t*-test was used to determine the significance of the change in pulmonary function and cervical alignment following diaphragmatic breathing and cervical joint mobilization. The data are presented as means and standard deviations. The statistical significance level was set at α = 0.05, a two-tailed test.

## 3. Results

### 3.1. General Characteristics of Subjects

Twenty participants who performed diaphragmatic breathing with cervical mobilization were analyzed in this study. The results of the analysis of the general characteristics of the study participants are shown in Table 1. The subjects included 14 men and 6 women, and the paralysis was right hemiparesis and left hemiparesis in 12 and 8 patients, respectively. The average disease duration of patients was 11.50 ± 3.33 months, the average age of patients was 64.25 ± 11.47 years, the average height was 165.68 ± 8.35 cm, the average weight was 64.86 ± 9.43 kg, the average BMI was 23.51 ± 1.90 kg/m^2^, the average K-MMSE was 25.70 ± 1.78 points, and the average K-NIHSS was 10.00 ± 3.48 points.

### 3.2. Changes in Pulmonary Function, Craniovertebral Angle

The effects of diaphragmatic breathing with cervical joint mobilization on pulmonary function and craniovertebral angle in subjects are shown in Table 2. FEV1 increased from 2.15 ± 0.62 ℓ before intervention to 2.33 ± 0.64 ℓ after intervention (*p* < 0.01); FVC increased from 2.64 ± 0.55 ℓ before intervention to 2.91 ± 0.64 ℓ after intervention (*p* < 0.01); PEF increased from 213.30 ± 88.52 ℓ/min to 254.00 ± 98.34 ℓ/min (*p* < 0.01); and FEV1/FVC changed from 80.00 ± 9.96% to 79.00 ± 8.51% (*p* > 0.05). The craniovertebral angle increased significantly, from 45.39 ± 6.26° before intervention to 46.04 ± 6.14° after intervention (*p* < 0.01).

## 4. Discussion

In this study, we applied diaphragmatic breathing with cervical joint mobilization to patients with stroke to assess whether they improved pulmonary function and the craniovertebral angle. Our findings were consistent with a previous study, showing that the cervical spine angle improved after cervical joint mobilization [32]. In addition, this study was able to confirm an increase in respiratory function.

Patients with stroke often sit in a slumped position due to poor postural control [15]. In addition, having to always use a wheelchair leads to a worse posture; the patients with stroke participating in this study were all hospitalized who relied on wheelchairs for mobility [33]. When a slumped position is maintained, kyphosis occurs, and the lumbar spine appears flat [15]. This eventually leads to forward neck posture and changes the alignment of the cervical vertebra [15]. Abnormal alignment of the cervical vertebra can capture or block the path of the spinal or phrenic nerve extending from the vertebrae [16,17,18]. Manual therapy of the C3 vertebra increased the maximum inspiratory pressure and maximum expiratory pressure in healthy people [23,34]. However, prior studies were conducted in healthy people, and manual therapy was applied to cervical vertebra manipulation [23,34]. Manipulation is a fast application method with high velocity and low amplitude; thus, there is a disadvantage in that the patient cannot stop before feeling abnormal [35]. In this study, cervical joint mobilization was applied that allowed patients to stop the movement at any time when they experienced an abnormality. Cervical joint mobilization applied to stroke patients immediately reduces muscle tone and stiffness [20]. In previous studies, this was the result of the expansion of the spinal nerve originating from the vertebrae through re-alignment of the cervical vertebra. Therefore, it is believed that joint mobilization applied to the cervical vertebra promotes the phrenic nerve and increases diaphragm contraction by affecting the activity of the descending pathway. Cervical spine correction can improve postural alignment, which can increase rib movement.

Patients with a forward neck posture have limitations in the movement of the rib cage [36]. Joint mobilization applied to the cervical and thoracic spine increases pulmonary function through increasing rib cage movement by re-aligning the vertebrae [37]. The joint mobilization site applied in this study was also a C3-C4 cervical vertebra; therefore, it was hypothesized that it affected the re-alignment of the vertebrae and had a positive effect on respiratory function through increased rib cage movement. In this study, while applying cervical joint mobilization, the patient was asked to maintain awareness of their respiration. The recognition of respiration in this study is noteworthy in that it used diaphragmatic breathing [38]. The phrenic nerve is located at C3-C4 [39]. Cervical joint mobilization activated the phrenic nerve through mechanical stimulation [23,28,34], and diaphragmatic breathing resulted in an immediate increase in the saturation of peripheral oxygen because diaphragmatic contractions were more activated [38].

Despite these results, this study has several limitations. First, because diaphragm movements and cervical joint mobilization were applied at the same time, it was not possible to determine which of the two had more influence on the respiratory function. Secondly, this study lacks internal and external validity because there was no control group due to the single-group pre–post design. Thirdly, the number of subjects in this study was very small, and the findings may not be generalizable to all stroke patients. Additionally, the short intervention period could have prevented interference with other treatments, but the long-term effects have not been confirmed. Finally, in this study, respiratory muscle strength could not be objectively confirmed. PEF has a strong correlation with maximal inspiratory pressure and maximal expiratory pressure, i.e., respiratory muscle strength [40,41]. This study measured the pulmonary function to check the flow of air because of diaphragmatic breathing, which is an inhalation and exhalation exercise. Even if PEF is strongly correlated with MIP and MEP, it is necessary to confirm direct respiratory muscle strength. In addition, various measures such as joint stiffness, ventilatory capacity measures, and perceptual measures are still needed to complete the results.

However, this study has clinical significance in that it is the first study to apply both diaphragmatic movement exercise and cervical joint mobilization. Joint mobilization is a safe method, and diaphragmatic exercise has the advantage that any physical therapist can utilize it. In addition, it is expected that follow-up studies will be conducted based on the effect size identified in this study. Nevertheless, the single group and small number of subjects remain a problem. This issue should be addressed by a randomized study design with a control group.

## 5. Conclusions

The purpose of this study was to investigate the effect of diaphragmatic breathing with cervical joint mobilization on pulmonary function and cervical alignment in 20 stroke patients. It was found that diaphragmatic breathing with cervical joint mobilization was effective in improving pulmonary function and cervical alignment in patients with stroke. However, based on this study, it is difficult to draw conclusions about the difference in value due to the absence of a control group. In future studies, it will be necessary to compare the effect of diaphragmatic breathing with joint mobilization with changes in a control group in a larger group of subjects.

## Figures and Tables

**Table 1:** General characteristics of the subjects.

	Experimental Group (*n* = 20)
Gender(male/female)	14/6
Affected side(left/right)	12/8
Pathogenesis (hemorrhage/infarction)	5/15
Disease duration (months)	11.50 ± 3.33
Age (years)	64.25 ± 11.47
Height (cm)	165.68 ± 8.35
Weight (kg)	64.86 ± 9.43
BMI (kg/m^2^)	23.51 ± 1.90
K-MMSE (points)	25.70 ± 1.78
K-NIHSS (points)	10.00 ± 3.48

All values are denoted as mean ± standard deviations. BMI: Body mass index. K-NHSS: Korean version of the National Institutes of Health Stroke Scale. K-MMSE: Korean version of mini-mental state examination.

**Table 2:** Changes in pulmonary function, craniovertebral angle after intervention.

Classification	Experimental Group (*n* = 20)		Within Group Change ^a^	*t*	*p*	%	Effect Size
	Pre-Test	Post-Test					
FEV1 (ℓ)	2.15 ± 0.62	2.33 ± 0.64	0.18 ± 0.19 (0.10, 0.27)	4.405	0.001 *	8.37	0.95
FVC (ℓ)	2.64 ± 0.55	2.91 ± 0.64	0.26 ± 0.22 (0.16, 0.37)	5.424	0.001 *	10.23	1.23
FEV1/FVC (%)	80.00 ± 9.96	79.00 ± 8.51	−1.00 ± 5.87 (−3.75, 1.75)	−0.762	0.455	−1.25	0.17
PEF (ℓ/min)	213.30 ± 88.52	254.00 ± 98.34	40.70 ± 46.85 (18.78, 62.62)	3.885	0.001 *	19.08	0.87
CVA (°)	45.39 ± 6.26	46.04 ± 6.14	0.64 ± 0.72 (0.31, 0.98)	4.031	0.001 *	1.43	0.90

All values are denoted as mean ± SD, ^a^ Values are the mean (95% CI). * *p* < 0.05. FEV1: Forced expiratory volume in 1 s. FVC: Forced vital capacity. PEF: Peak expiratory flow. CVA: Craniovertebral angle. Experimental group: Diaphragmatic breathing with joint mobilization.

## Data Availability

Study data are available upon request from corresponding author.
